# Determinants of virological failure among HIV clients on second-line antiretroviral treatment at Felege-hiwot and University of Gondar comprehensive specialized hospitals in the Amhara Region, Northwest Ethiopia: A case-control study

**DOI:** 10.1371/journal.pone.0289450

**Published:** 2024-07-09

**Authors:** Getahun Ayenew, Yeshambel Agumas, Tebkew Shibabaw, Gebremariam Getaneh, Michael Getie

**Affiliations:** 1 Department of Molecular Laboratory, Trachoma Elimination Program, The Carter Center Ethiopia, Bahir Dar, Ethiopia; 2 Department of Health System Management, Leadership Development Program, School of Public Health, College of Medicine and Health Science, Bahir Dar University, Bahir Dar, Ethiopia; 3 Department of Environmental Health, School of Public Health, College of Medicine and Health Science, Bahir Dar University, Bahir Dar, Ethiopia; 4 Department of Health Systems Management and Health Economics, School of Public Health, College of Medicine and Health Science, Bahir Dar University, Bahir Dar, Ethiopia; 5 Department of Medical Microbiology, Amhara National Regional State Public Health Institute, Bahir Dar, Ethiopia; Korea Disease Control and Prevention Agency, REPUBLIC OF KOREA

## Abstract

**Background:**

Second-line HIV treatment failure has become increasing worldwide, mainly in sub-Sahara Africa including Ethiopia. Even though the problem becomes increasing, inadequate information was available about its magnitude and associated factors in the current study area.

**Objective:**

To assess the factors of second-line Anti-Retroviral Treatment virological failure among second-line ART users.

**Method and materials:**

Institutional-based unmatched case-control study design was conducted from September to December 2021 at Felege Hiowt and University of Gondar Comprehensive Specialized Hospitals; Amhara region, Northwest Ethiopia. A total of 216 patients (60 cases and 156 controls) were recruited by a simple random sampling technique with a 1:3 cases-to-controls ratio. Patients who had two viral load results >1000 copies/ml within a 3-month interval after taking ART drugs for at least 6 months were cases and those who had ≤1,000 copies/ mL were controls. The sample size was calculated by using Epi-Info version 7.2.4. Structured questionnaires were used to gather the required information. SPSS version 26 was used to summarize the findings. In bivariate logistic regression model, Variables with two-tailed P-value ≤ 0.25 at 95% confidence interval were transferred into multivariate binary logistic regression model and P value at ≤ 0.05 was set as statistically significant.

**Results:**

Out of 216 patients recruited, 212 have participated with a response rate of 98.2%. From these participants, 117(55.2%) were males and 187(88.2%) were urban dwellers. Among the total respondents, 208(98.1%) had age > 24 years, 200(94.3) were at HIV clinical stage I, 72(34%) had poor ART adherence and 112(52.8) did not disclose their HIV status. Likewise, most of the patients 147(69.37) didn’t use condoms. The associated factors were not disclosing HIV status (AOR = 3.4, 95% CI: 1.52–7.79), medium adherence (AOR = 3.7, 95% CI = 1.3–10.7), poor adherence level (AOR = 5.27, 95% CI: 2.2–12.5), not using condoms (AOR = 4.47, 95% CI: 1.63–12.2) and Viral load (>150 copies/ml) when switched to second-line ART (AOR = 3.56, 95% CI: 1.5–8).

**Conclusion and recommendations:**

Non-disclosure, poor or medium adherence, not using condoms and high Viral load (>150 copes/ml) when switched to second-line ART were the main factors for second-line Anti-Retroviral Treatment virological failure. Disclosure about HIV status, using condoms and improving treatment adherence level are crucial to reduce second-line virological failure.

## Introduction

Human immunodeficiency virus (HIV) has continued to be a major global public health issue, having claimed almost 40.4 million lives so far. About 85.6 million people have become infected with HIV since the start of the epidemic. Even though HIV infection prevention, diagnosis, treatment and care has increased, the disease remains a major public health threat worldwide due to the emergence of drug-resistant HIV [1, 2]. All current antiretroviral drugs (ARVD), including newer classes, are at risk of becoming partly or fully inactive [3, 4]. This results an increase in mortality, morbidity, HIV incidence and health care costs [5].

Viral load failure is used as a golden approach to confirm antiretroviral therapy (ART) failure. For HIV patients who started ART, Viral load testing should be performed early after initiating ART within 6 months, at 12 months and then at least every 12 months to detect treatment failure [5]. If a person on ART who has taken second-line ART drugs for at least 6 months has two consecutive viral load (Vl) results greater than 1000 copies/ml within a 3-month interval with adherence support between measurements, the results will confirm failure of the current treatment regimen and the client needs to be switched to appropriate third line regimen [5, 6].

Globally, the prevalence of drug resistant HIV (DRHIV) to non-nucleoside reverse transcriptase inhibitor (NNRTI) drugs has significantly increased since 2001. In Sub-Saharan Africa (SSA), Second-line HIV treatment failure has become highly prevalent with alarming rates during the 12–18-month period of treatment starts [7, 8]. Among patients receiving protease inhibitor (PI) based 2nd-line HIV treatment, 25% experienced virological failure at some point during follow-up [9]. A systematic review and meta-analysis conducted in SSA in 2019 also reported that the prevalence of Second-line HIV treatment failure was 13.4%. Of which 65% were PI-based and 35% were Ritonavir boosted PI-based second-line ART regimens [7]. The problem is observed more rapidly in Eastern Africa with an estimated annual incremental increase of 29%, Southern Africa (23%), Western and Central Africa (17%), Latin America (15%), and Asia (11%). In eastern Africa, the Prevalence of pretreatment NNRTI resistance increased from 0% in 1996 to 13% in 2016 [7, 10, 11]. In resource-limited countries, HIV drug resistance is still a serious threat to their health [3]. The prevalence of second-line virological ART failure rates among adults in 2012 in these country were 21.8–33%, 14–38%, 15–38.5% and 10–38.0% at 6, 12, 24, and 36 months of initiation on second-line ART respectively [12]. There were 1668 patients with virological failure in south Africa in 2012 [13], 109 patients with confirmed second-line virological failure in Malawi in 2010 [14]. Second-line ART Virological failure was 18% in Rwanda by the end of 2016 [15]. Some Studies in Tanzania and southeast Uganda also indicated that there were high incidence of virological failure on second-line ART [16, 17].

The magnitude of virological treatment failure is increasing from time to time in Ethiopia [18]. It becomes a major challenge for HIV AIDS prevention and control mechanisms. The adjusted magnitude of second-line virological failure (VF) among the population taking ART in Ethiopia was found to be 11% [19]. According to a study done among children and adolescents on second line ART in a pediatric cohort of an Ethiopian tertiary hospital, the proportion of treatment failure (TF) was 14 out of 76 patients [20]. A high incidence rate of second-line treatment failure (9.86 per 100 person-years or 254/1011) was also noticed in Amhara Region in 2016 [21].

Among the determinants of second-line ART failure, poor adherence to ART, high viral load, not disclosure about HIV status, opportunistic infection, low CD4 counts < 350 cell/mm^3^, low BMI (< 16 kg/m^2^), young age (15–29 years old) patients, WHO HIV clinical stage, TB co-morbidity, smoking and drug abuse were found to be the main factors [7, 15, 21–26].

Even if the problem is rising, there is still a dearth of information in developing countries on the factors for second-line ART failure [14]. Data available on this regard is limited in the current study area. Hence, identifying the risk factors of second-line treatment failure stays crucial to reduce treatment failure on this regimen and the need for third-line drugs [27]. Most of the previous studies conducted in Ethiopia were focused on first-line ART drug resistance. Despite few investigations were done in this field, clinical and immunological characteristics of the patient were used to rule out ART failure, which is not recommended whenever viral load monitoring is possible. Therefore, identifying the factors of second-line ART virological failure among patients on ART was the main objective of this study.

Findings of this research will give information about the risk factors of second-line ART virological failure in the current study area. It will serve as a base line data for treatment centers to address factors related to second line treatment failure. The result might be useful for stakeholders to set programs and action plans on the need to third line regimens. It will be used as a source of information for researchers. It will also give important recommendations for patients on their treatment.

## Methods and materials

### Study settings

The study was conducted at Felegehiwot and University of Gondar comprehensive specialized hospitals in the Amhara region, Northwest Ethiopia. Felegehiwot Comprehensive Specialized Hospital (FHCSH) is found in Bahir Dar city, the capital city of Amhara Regional state. It is located 567km away from Addis Abeba. According to the Amhara National Regional State Plan Commission Bureau 2021 data, Bahir Dar city administration had an estimated total population of 412,189 (206,412 male and 205,777 female) dwellers. Of which 85% live in the urban and 15% in the peri-urban and rural areas of the city [28, 29]. Based on Amhara Regional health bureau 2021 report, 14 Health posts, 10 Health centers 2 general hospitals and 2 referral hospitals are governmental health institutions found in the city. There are also 10 basic clinics, 28 Medium clinics, 10 higher clinics and 5 general hospitals among private health institutions. Only one governmental specialized referral hospital (Felege Hiwot) has provided third line ART treatment service in Bahir Dar [30]. There were 740 and 31 ART patients who were on second line and third-line regimens respectively at FHCSH by the end of August 2021.

The city of Gondar is situated in North-western parts of Ethiopia, Amhara Regional State. It far 725 km from Addis Ababa, 175 km from Bahir Dar and 120km from the Simien Mountains. The Regional Plan Commission Bureau also reported that, the metro area population of Gondar city in 2021 was 454,445 (218,378 male and 236,068 female) [29, 31]. University of Gondar Comprehensive Specialized Hospital (UGCSH) is used as the referral center for more than 7 million catchment population [32]. Based on reports obtained from this hospital, there were 5513 patients on ART until September 1^st^, 2021. Out of these 520 were second-line ART users and 29 were on third-line regimens.

### Study design and period

Institutional-based unmatched case-control study design was conducted from September first, 2021, to December last 2021.

### Source population

All HIV patients at FHCSH and UGCSH who were on second-line ART regimens.

### Study population

All HIV patients on ART at FHCSH and UGCSH who took second line ART for more than six months and had two subsequent viral load measurements within a 3-month interval during the study period.

### Inclusion and exclusion criteria

All HIV patients who were on second-line ART at FHCSH and UGCSH ART clinics during the study period were included. Whereas those patients who had not taken a second-line ART regimen for more than six months and who didn’t have two subsequent viral load measurements within a 3-month interval during the study period were excluded from the study.

### Sample size determination and sampling techniques

Sample size was calculated using EPIINFO version 7.2.4 (a two-population proportion formula). The following assumption were considered during sample size calculation. Confidence level 95%, Z α/2 = 1.96 (for 0.05 significance level); for 80% power, Z_β_ = 0.84, ratio of controls to cases = 3:1, p1 = 31% (% of cases among exposed), p2 = 11.2% (% of cases among non-exposed). Key predictors of second-line ART virologic failure from a previous study carried out in Wollo, Northeast Ethiopia, were used a base line data [26]. Hence, the minimum sample size including 10% non-respondent rate for this study was 216 participants, 60 cases and 156 controls. The list of clients with their viral load results were extracted from the patients registration book in each ART clinic of study facilities. Then 31 patients at FHCSH and 29 patients at UGCSH who had a viral load result >1,000 copies/mL in two subsequent viral load measurements within a 3-month interval were selected. Likewise, 94 patients from FHCSH and 62 patients from UGCSH who didn’t develop a virological failure were recruited based on proportional allocation by using a computer generated simple random sampling method.

### Study variables

Independent variables were Socio demographic, behavioral, clinical, and immunological characteristics of the patient. Of these; sex, age, residence, marital status, educational status, alcohol intake, drug abuse, smoking, disclosure status, condom use, adherence, body mass index (BMI), opportunistic infections (OI), TB coinfection, nutritional status, WHO HIV clinical staging at switch to second line, CD4 count (CD4 count at 2^nd^ line ART initiation), viral load, type of first and second line treatment (being on a protease inhibitor plus 2 nucleoside analogues(NRTIs) or not), duration in second line ART and duration in first line ART. Whereas the dependent variable (outcome of interest) was second line ART virological failure.

### Data collection tools and techniques

The data were collected by using structured questionnaires and checklist prepared by the principal investigator. The tools were adapted from different literatures, WHO guidelines, institutional checklists, and some of it were developed by the investigators. This tool includes socio demographic, behavioral, clinical, and immunological related data. Personal interview and patient registration book/ card/ review was performed using data collection tools to gather the required information.

### Data quality managements

To maintain the data quality, different techniques were employed to address major source of errors. The questionnaires were translated to local language (Amharic). Health professionals who were participated in the data collection were got on sight training before the data collection. Each day the collected data were cross checked for its completeness, accuracy, clarity, consistency and missed values and missed variables carefully. Necessary modifications were made based on the gaps identified. All collected data were entered into EPI-data version 3.1 after being coded. Then it was transferred to Statistical Package for Social Science (SPSS) version 26 for analysis.

### Data processing and analysis

The collected data were entered and analyzed by using SPSS version 26 for windows. Descriptive statistics was used to present and summarize the findings. Bivariate and multivariate binary logistic regression model was employed to identify determinants of second line ART virological failure. Odds ratios with its 95% confidence intervals and two-tailed P-value was calculated. Variables with P-value ≤ 0.25 in the bivariate analysis were included in the multivariate logistic regression model and variables that had a P value ≤ 0.05 in the multivariate analysis were considered statistically significant.

### Ethical statement

This investigation was approved by Bahir Dar University, College of Medicine and Health Sciences Institutional Review Board (P. No-317/2021). Official letter of co-operations was provided to FHCSH and UGCSH prior to data collection. Assent from children and verbal informed consent from adults were obtained after explaining the purpose and objective of the study. Confidentiality of the data had been maintained.

## Definition of terms

**Stable on ART-** HIV Patients on ART for at least 1 year, no current illnesses or pregnancy, good understanding of lifelong adherence and evidence of treatment success (two consecutive viral load measurements below 1000 copies/mL) and absence of adverse drug reactions requiring regular monitoring [5].

**Adherence**- Refers to the whole process from starting HIV treatment, keeping all medical appointments and taking HIV medicines every day and exactly as prescribed. Or it is the degree to which the person’s behavior, taking medication, following a diet and/or changing lifestyle corresponds with the agreed recommendations from a health care provider [5].

**Good Adherence;** drug adherence of ≥95% or if <2 doses of 30 doses or <3 doss of 60 doses is missed as documented by ART healthcare provider [5].

**Fair/medium/ adherence**. Drug adherence of 85–94% or 3–5 missed drug doses out of 30 doses or 4–9 missed drug doses out of 60 doses [5].

**Poor adherence;** If drug adherence is <85% or ≥6 doses of missed ART drug doses out of 30 doses or >9 doses of missed ART drug doses out of 60 as documented by the ART healthcare provider [5].


$$\mathrm{Adherence}\;\mathrm{index}=\frac{\mathrm{Total}\;\mathrm{number}\;\mathrm{of}\;\mathrm{drugs}\;\mathrm{taken}}{\mathrm{Total}\;\mathrm{number}\;\mathrm{of}\;\mathrm{drugs}\;\mathrm{prescribed}}\times 100$$

**Suboptimal adherence**; a report of ≥1 reason for missing ART ≥5 times within the past month [33]

**Opportunistic Infections (OI);** Infections that develop as a result of HIV-inflicted damage to the immune system (TB, PCP, gastrointestinal OI, herpes simplex, herpes zoster, fungal infection) and other national ART guidelines define opportunistic infection [6].

**Virological failure;** refers to a persistently detectable viral load exceeding 1000 copies/mL (two consecutive viral load measurements within a 3-month interval with adherence support between measurements) after at least 6 months of using ART [5]

**Non-Disclosure:** patients who did not disclose about their HIV status for anyone except their health care providers who give the service.[5]

## Results

### Socio-demographic and behavioral characteristics

Out of the total of 216 patients on second-line ART (60 cases and 156 controls) recruited, 212 (59 cases and 153 controls) were participated in this study. This accounts for a response rate of 98.2%. The mean age of the participants were 40.7 years old. Among the participants, 117 (55.2%) were males and 187 (88.2%) respondents were urban residents. The majority of respondents 208 (98.1%) were above 24 years old, and only 4 (1.9%) were 24 years old or below. There were 102 (48.1%) married participants and 73 (34.4%) who had an elementary level of education (Table 1).

**Table 1:** Socio demographic characteristics of patients on second-line ART at FHCSH and UGCSH; Amhara Region, Northwest Ethiopia from September to December 2021.

General variables	Variables category	Frequency of Virological failure. No. (%)		Total No. (%)
		Cases (N = 59) No. (%)	Controls (N = 153) No. (%)	N = 212
Sex	Male	33(55.9)	84(54.9)	117(55.2)
	Female	26(44.1)	69(45.1)	95(44.8)
Age (in year)	< 14	3(5.1)	1 (0.7)	4(1.9)
	14–24	4(6.8)	13(8.5)	17(8.1)
	>24–64	51(86.4)	136(88.9)	187(88.2)
	>64	1(1.8)	3(2)	4(1.9)
Residence	Urban	51(86.4)	136(88.9)	187(88.2)
	Rural	8(13.6)	17(11.1)	25(11.8)
Educational status	Illiterate	14(23.7)	43(28.1)	57(26.9)
	Elementary level	21(35.6)	52(34)	73(34.4)
	Secondary level	12(20.3)	21(13.7)	33(15.6)
	Preparatory level	11(18.6)	20(13.1)	31(14.6)
	College and above	1(1.7)	17(10)	16(7.5)
Marital status	Single	16(27.1)	48(31.4)	64(30.2)
	Married	34(57.6)	68(44.4)	102(48.1)
	Divorced	7(11.9)	25(16.3)	32(15.1)
	Widowed	2(3.4)	12(7.8)	14(6.6)

From the study participants, 33 (55.9%) of the cases and 3(2%) of the controls had poor adherence to ART treatments. Similarly, 46 (78%) of the cases and 66 (43.1%) of the controls did not disclose their HIV status while taking ART. Likewise, 9 (15.3%) of the cases and 26 (17%) of the controls had a history of alcohol use. Most of the cases 53 (89.8%) and the controls 94 (61.4%) did not used condoms (Table 2).

**Table 2:** Behavioral characteristics of patients on second-line ART at FHCSH and UGCSH; Amhara Region, Northwest Ethiopia from September to December 2021.

General variables	Variables category	Frequency of Virological failure. No. (%)		Total No. (%)
		Cases (N = 59) No. (%)	Controls (N = 153) No. (%)	212
Alcohol consumption	Yes	9(15.3)	26(17)	35(16.5)
	No	50(84.7)	127(83)	177(83.5)
Using condom	Yes	6(10.2)	59(38.6)	65(30.7)
	No	53(89.8)	94(61.4)	147(69.374.5)
Disclosure status	Disclosed	13(22)	87(56.9)	100(47.2)
	Not disclosed	46(78)	66(43.1)	112(52.8)
Smoking cigarettes	Yes	5(8.5)	1(0.7)	6(2.8)
	No	54(91.5)	152(99.3)	206(97.2)
Level of Adherence	Good	13(22)	91(59.5)	104(49)
	Medium	13(22)	39(25.5)	36(17)
	Poor	33(55.9)	3(2)	72(34)

### Clinical and immunological characteristics

Among the participants recruited, 86(40.6%) had a CD4 count <200 (cells/mm3). Eight (13.6%) of the cases and 7(4.6%) of the controls were severely malnourished. Viral load when switched to second-line ART is ≥1000(copes/ml) for 71(33.5%) patients. Most of the participants were at WHO HIV clinical stage I (Table *3*).

**Table 3:** Clinical and immunological characteristics of patients on second-line ART at FHCSH and UGCSH; Amhara Region, Northwest Ethiopia from September to December 2021.

General variables	Variables category	Frequency of Virological failure. No. (%)		Total No. (%) N = 212
		Cases (N = 59) No. (%)	Controls (N = 153) No. (%)	
Body mass index (BMI)	<18.5	20(33.9)	25(16.3)	45(21.2)
	18.5–25	36(61)	106(69.3)	142(67)
	>25–30	2(3.4)	21(13.7)	23(10.8)
	>30	1(1.7)	1(0.7)	2(0.9)
CD4 count (cells/mm3)	<200	24(40.7)	62(40.5)	86(40.6)
	200–350	21(35.6)	53(34.6)	74(34.9)
	350.01–500	9(15.3)	29(19)	38(17.9)
	>500	5(8.5)	9(5.9)	14(6.6)
Nutritional status	Normal	33(55.9)	104(68)	137(64.6)
	MAM (Moderately)	10(16.9)	25(16.3)	35(16.5)
	SAM (severely)	8(13.6)	7(4.6)	15(7.1)
	Overweight	8(13.6)	17(11.1)	25(11.8)
Co-trimoxazole started	Yes	28(47.5)	74(48.4)	102(48.1)
	No	31(52.5)	79(51.6)	110(51.9)
Fluconazole started	Yes	3(5.1)	22(14.4)	25(11.8)
	No	56(94.9)	131(85.6)	187(88.2)
Viral load when switched to second line ART (copes/ml)	<150	18(30.5)	104(68)	122(57.5)
	150–999.999	11(18.6)	8(5.2)	19(9)
	≥1000	30(50.8)	41(26.8)	71(33.5)
WHO HIV clinical stage	stage I	49(83.1)	151(98.7)	200(94.3)
	stage II	1(1.7)	1(0.7)	2(0.9)
	stage III	9(15.3)	1(0.7)	10(4.7)
Duration in first line ART /in years/	<2 years	6(10.2)	26(17)	32(15.1)
	2–5 years	16(27.1)	58(37.9)	74(34.9)
	5.01–10 years	30(50.8)	60(39.2)	90(42.5)
	> 10 years	7(11.9)	9(5.9)	16(7.5)

MAM- Moderate Acute Malnutrition, SAM: Severe Acute Malnutrition

### Factors associated to second line ART virological failure

In bi-variable logistic regression analysis the following variables; Not using condoms, Non-disclosure about HIV status, poor Level of Adherence, sever acute malnutrition nutritional status and Viral load >150 copes/ml when switched to second line ART were selected for further multivariable binary logistic regression analysis (P-value<0.25) (Table 4).

**Table 4:** Factors associated to second line ART virological failure among second-line HIV patients at FHCSH and UGCSH; Amhara Region, Northwest Ethiopia from September first, 2021, to December last 2021.

General variables	Variables category	Virological failure		Odd Ratios			
		Yes	No	COR (95% CL)	P- value	AOR (95% CL)	P- value
Using condom	Yes	6	59	1			
	No	53	94	5.54 (2.3–13.7)	0.000	4.5(1.63–12)	0.004
Disclosure status	Disclosed	13	87	1			
	Not disclosed	46	66	4.6(2.33–9.34)	0.000	3.4(1.5–7.8)	0.003
Level of Adherence	Good	13	91	1			
	Medium	13	23	3.95(1.6–9.7)	0.003	3.7(1.3–10.7)	0.014
	Poor	33	39	5.9(2.8–12.4)	0.000	5.3(2.2–12.5)	0.000
Nutritional status	Normal	33	104	1			
	MAM	10	25	1.26(0.55–3)	0.58	1.2(0.4–3)	0.75
	SAM	8	7	3.6(1.2–10.7)	0.021	3.56(1–14.4)	0.075
	Overweight	8	17	1.48(0.6–3.8)	0.045	1.6 (0.4–5)	0.46
Viral load when switched to second line ART (copes/ml)	<150	18	104	1			
	150– <1000	11	8	7.9(2.8–22)	0.000	5.4(1.5–19)	0.009
	≥ 1000	30	41	4.2(2.1–8.4)	0.000	3.56(1.5–8)	0.002

In the multivariable binary logistic regression analysis, Not using condoms, non-disclosure about HIV status, poor or medium level of adherence and viral load ≥150 copes/ml when switched to second line ART had a significant impact on second line ART outcomes. Peoples who did not disclosed their HIV status were 3 times (AOR = 3.4, 95% CI: 1.52–7.79) more likely to develop second-line ART virological failure as compared to those patients who did. Among the respondents those patients who had poor and medium level of adherence were 5 times (AOR = 5.27, 95% CI = 2.2–12.5) and 3.7 times more likely to develop second line ART virological failure respectively as compared to those who had a good adherence (Table 4).

Moreover, the likelihood of developing second line virological failure among patients with Viral load ≥1000 copes/ml when switched to second-line ART medication were 3.5 times (AOR = 3.56, 95% CI: 1.5–8) more as compared to those patients with Viral load result <150 copes/ml. Similarly, those patients with VL>150–999 copies/ml had 5 times more to develop virological failure as compared to those with VL<150 copies /ml. (Table 4).

## Discussion

This study was aimed to assess factors for virological failure among second-line ART users. Poor or medium level of adherence to ART, non-disclosure about HIV status, not using condoms and having Viral load >150 copes/ml when switched to second line ART were the factors that increased the odds of second line ART virological failure.

Patients who had poor and medium ART adherence were 5 times and 3.7 times more likely to develop second line ART virological failure as compared to patients who had good treatment adherence. Similar findings were reported in Wollo, Amhara Regional State Northeast Ethiopia and Northern Ethiopia where the odds of developing second line ART virologic failure was strongly associated with poor ART adherence [22, 34]. This finding is supported by a study conducted in Johannesburg, South Africa [27], Southwestern Nigeria [35], sub-Saharan Africa and Asia which showed that poor or medium adherence was a strong predictor of second-line ART failure [7, 23]. Another investigation done in Cambodia (Southeast Asian) also confirmed that, patients on second line ART with good adherence were significantly associated with viral suppression [24].

ART adherence is generally regarded as an important factor in achieving optimal treatment outcomes. High levels of treatment adherence to ART results to effective viral suppression outcomes. Poor adherence to ART is the most frequent cause of treatment failure and the subsequent development of drug resistant strains of HIV. If the patients do not always take their medications as prescribed, the virus will replicate and develop new resistance mutations over time. Missing doses can lead to low drug levels in the body, which allows the virus to resume replication and accumulate resistance mutations as it multiplies. This results less effective viral suppression and a rise in plasma viral load. Inadequate suppression of viral replication has a continued destruction of CD4 cells, progressive decline in immune function and disease progression, Limited future treatment options and higher costs to the individual and ARV program. Even though these risks the immediate health of the patient, it is also an important reason for the emergence of viral resistance to one or more antiretroviral medications [36, 37].

The study found that, not using condom was one of the factors for second line ART virological failure. Patients who didn’t use condom were 4 times more likely to develop ART failure than those who used.

Inconsistent use of a condom by people living with HIV/AIDS on ART has led to further risk of new HIV infection and the development of reinfection with new drug-resistant viral strains. Using condom prevents from acquiring and transmitting drug resistant HIV infection. Patients on ART may also acquire transmitted drug resistant HIV virus if they do not use condoms. This type of HIV infection results treatment failure among ARV drug–naive people with no history of ARV drug exposure [3, 38]. Not using Condom also exposed for many sexually transmitted infections (STI) that leads to further worsening the HIV infection by dipping the immune system. So that ART may not be effective and new drug resistant viral strains may occur [39].

The study also identified that patients who had a high viral load (≥1000 copies/ml) when switched to second line ART were 3.5 times more likely to develop second-line ART virological failure than those patients who had suppressed viral load (<150 copies/ml). This outcome is supported by studies conducted in Sub-Saharan Africa and Johannesburg, South Africa in which they presented that high viral load was a significant predictor for second line ART virological failure [7, 27].

Different literature stated that a high viral load can lead to a low CD4 cell count which in turn increases the risk of developing an illness or infection. Circulating high viral particles in blood results high destruction of the immune system (CD4 cell) which in turn aggravates disease progression. Finally, it will lead to the emergence of resistant viral strains. This indicates the treatment is not working well. The higher the viral load, the more risk developing treatment failure [40].

In addition, our investigation identified that the odds of developing second line virological ART failure was increased by 3 folds for patients who did not disclose their HIV status as compared to their counter parts.

Previous studies conducted in Wollo, Amhara Regional State, Northeast Ethiopia and Johannesburg, South Africa also reported that not disclosing HIV status was the main risk factors for virological ART failure [27, 34]. But a study conducted in a post-conflict area in Northern Uganda have reported its impact on treatment outcomes. It showed that HIV-infected people have got confronted discrimination, stigmatization, and violence following their disclosure. This may be patients in conflict-afflicted areas may in specific confront exceptional challenges, and their need for social support may be aggravated [41].

Most studies had agreed that disclosure play a significant role in good ART outcomes [42]. People who achieved full disclosure about their HIV status had lower viral load (improved viral suppression), increased ART adherence and allowed for better self-care and treatment. Patients who disclosed about their HIV status have good adherence to ART compared with those who did not. Studies have found that patients who disclosed their sero-status had better social support; stronger family and relationship cohesion; reductions in anxiety and depression; improvements in physical health, emotional support, and financial support; and were better able to take their ART freely. Talking about having HIV may lead to increased trust and feelings of intimacy in a relationship or close friendship and a feeling of empowerment. This helps to have a higher chance of being supported by loved ones and partners. For example, disclosing for health professionals such as doctor, dentist, pharmacist, or psychologist will mean that they are better able to provide appropriate care that meets the patients’ needs as a person living with HIV. These helps to improve their ART adherence and having lower risk of developing ART failure. Patients who did not disclosed their HIV status were at high risk of developing Virological failure [43–45].

Although disclosure comes with a sense of alleviation and potential support, it also bears challenges like stigma, discrimination, and violence. Such perceptions make disclosure is not easier. To address this barrier, different methods are recommended. HIV care providers and counsellors should be trained by emphasising the positive outcomes of disclosure and provide frequent counselling service. They also prepare their clients to handle the potential negative outcomes and raise their awareness about the benefits of HIV disclosure. Selecting targets to be disclosed for the first time based on relationship quality and target opinions helps to reduce psychological stress and to adapt disclosure. Timing of disclosure should be carefully planned to minimize rejection and maximize fairness to others. Patients should be ready enough to overcome side effect of disclosure before they do to reduce the possible challenges [42, 46].

## Conclusions

The study identified that poor or medium adherence to ART medication, non-disclosure about HIV status, not using condom and high viral load >150 copies/ml were significantly associated with second-line ART virological failure.

### Recommendations

Patients on ART should not miss their medication for any reasons and use their full efforts to have good ART adherence. Disclosing their HIV status and using condom will have an incredible benefit in terms of social supports and to get early ART treatment. Early identification of patients who had poor adherence, non- disclosed and high viral load will be an important task to reduce ART failure. Such patients will need closer clinical follow-up to minimize the risk of treatment failure. Further investigation is needed to explore factors associated with poor adherence and not disclosing HIV status.

## Supporting information

S1 File Annex I. Information sheet. (DOCX)

S2 File Annex II Consent form. (DOCX)

S1 Table Socio demographic characteristics of the patients. (DOCX)

S2 Table Behavioral characteristics of the patients. (DOCX)

S3 Table Clinical and immunological features of the patients. (DOCX)

S4 Table Factors for second line ART Virological failure. (DOCX)
