# Supplementary material for: Determinants of virological failure among HIV clients on second-line antiretroviral treatment at Felege-hiwot and University of Gondar comprehensive specialized hospitals in the Amhara Region, Northwest Ethiopia: A case-control study
Source: PLoS One. 2024 Jul 9;19(7):e0289450. doi: 10.1371/journal.pone.0289450 (PMC11232969; doi:10.1371/journal.pone.0289450)
Supplement: S1 File — (DOCX) [file pone.0289450.s001.docx]

**Annex I****. Information sheet**

Deep greetings, my name is …………….

I temporarily represent Bahir Dar University, College of Medicine and Health Science, School of Public health.

Department of Health System Management and Health Economics. Here the mission justified below why I am here.

**Title of the research project:** Determinants of virological failure among HIV clients on second-line antiretroviral treatment at Felege-hiwot and University of Gondar comprehensive specialized hospitals in the Amhara region, Northwest Ethiopia: a case-control study.

**Name of Investigators:** Getahun Ayenew Wobetu, Yeshambel Agumas, Tebkew Shibabaw and Gebremariam Getaneh.

**Name of the Organizations**: Bahir Dar University

**Purpose of the study:** To determine the factors associated to virological ART failure among patients on second-line ART regimen at Felege Hiwot and university of Gondar Comprehensive specialized referral hospital. The study will provide base line information for concerned bodies and for further research.

**Procedure:** To perform this study, you are being one of the invited persons as you are fulfilled the criteria. If you are willing to participate, you need to understand the purpose of the study and give your consent verbally. Socio-demographic, socio-economic and clinical pattern information will be collected from the patients registration book using structured questionnaires. if there is a missed Socio-demographic or socio-economic data in the registration book, you are kindly asked to genuinely answer the prepared questionnaires.

**Potential risk factors**: the study has no risk for both the participants and the community.

**Benefits of the study:** this study will give some clues about the factors that contributes for second-line virological ART failure and to work against it.

**Compensation for participation**: You will not receive any payment for your participation in this study.

**Confidentiality of your information**- All information gathered from the study participant will remain confidential. If you do not need the result, your participation in this study can be anonymous. Personal information will be treated confidentially and under no circumstances will it be transmitted to any person or organization.

**Right to Refusal or Withdraw**: participating in this study is absolutely dependent on your willingness. You have a full right to refuse from participation as well as to cancel after you start at any course of the study. You can refuse to respond any or all the questionnaires and this will not affect the service you get from.

**Person to Contacts:** If you want to know more information about this study, you can contact the following organization and individuals at any time.

Organization - Bahir Dar University, Department of Department of Health System Management and Health Economics.

P.Box 79, Bahir Dar, Ethiopia

Individuals - Getahun Ayenew Wobetu

Phone no. - 0918512275

E-mail - [Getahun.ayenew@yahoo.coom](mailto:Getahun.ayenew@yahoo.coom)

Final participant decision: -  Agree to participate 

Not Agree to participate/refuse/ 
