# Supplementary material for: Determinants of virological failure among HIV clients on second-line antiretroviral treatment at Felege-hiwot and University of Gondar comprehensive specialized hospitals in the Amhara Region, Northwest Ethiopia: A case-control study
Source: PLoS One. 2024 Jul 9;19(7):e0289450. doi: 10.1371/journal.pone.0289450 (PMC11232969; doi:10.1371/journal.pone.0289450)
Supplement: S2 File — (DOCX) [file pone.0289450.s002.docx]

**Annex II Consent form**

Name of health institution--------------------------------------Code of the Participant --------- Date-------

I the study participant have been well informed about the objective of the study entitled on Determinants of virological failure among HIV clients on second-line antiretroviral treatment at Felege-hiwot and University of Gondar comprehensive specialized hospitals in the Amhara region, Northwest Ethiopia: a case-control study. And I am clear on this.

I clearly know that all the information obtained at any course of the study will be kept confidential. I also well informed of my right to keep hold of, decline to cooperate and drop out of the study if I don’t want and none of my actions will have any bearing at all on the service I get.

I agreed voluntarily to answer the provided questionnaires.

I disagreed to answer the provided questionnaires.

Name and signature of investigator /data collector/ …….……………Date……………….
