# Supplementary material for: Determinants of virological failure among HIV clients on second-line antiretroviral treatment at Felege-hiwot and University of Gondar comprehensive specialized hospitals in the Amhara Region, Northwest Ethiopia: A case-control study
Source: PLoS One. 2024 Jul 9;19(7):e0289450. doi: 10.1371/journal.pone.0289450 (PMC11232969; doi:10.1371/journal.pone.0289450)
Supplement: S1 Table — (DOCX) [file pone.0289450.s003.docx]

Table 1: Socio demographic characteristics of patients on second-line ART at FHCSH and UGCSH; Amhara Region, Northwest Ethiopia from September to December 2021.

| General variables | Variables category | Frequency of Virological failure. No. (%) | | Total No. (%) |
| --- | --- | --- | --- | --- |
|  |  | Cases (N=59)  No. (%) | Controls (N=153)  No. (%) | N = 212 |
| Sex | Male | 33(55.9) | 84(54.9) | 117(55.2) |
|  | Female | 26(44.1) | 69(45.1) | 95(44.8) |
| Age (in year) | < 14 | 3(5.1) | 1 (0.7) | 4(1.9) |
|  | 14 – 24 | 4(6.8) | 13(8.5) | 17(8.1) |
|  | >24 – 64 | 51(86.4) | 136(88.9) | 187(88.2) |
|  | >64 | 1(1.8) | 3(2) | 4(1.9) |
| Residence | Urban | 51(86.4) | 136(88.9) | 187(88.2) |
|  | Rural | 8(13.6) | 17(11.1) | 25(11.8) |
| Educational status | Illiterate | 14(23.7) | 43(28.1) | 57(26.9) |
|  | Elementary level | 21(35.6) | 52(34) | 73(34.4) |
|  | Secondary level | 12(20.3) | 21(13.7) | 33(15.6) |
|  | Preparatory level | 11(18.6) | 20(13.1) | 31(14.6) |
|  | College and above | 1(1.7) | 17(10) | 16(7.5) |
| Marital status | Single | 16(27.1) | 48(31.4) | 64(30.2) |
|  | Married | 34(57.6) | 68(44.4) | 102(48.1) |
|  | Divorced | 7(11.9) | 25(16.3) | 32(15.1) |
|  | Widowed | 2(3.4) | 12(7.8) | 14(6.6) |
