# Supplementary material for: Determinants of virological failure among HIV clients on second-line antiretroviral treatment at Felege-hiwot and University of Gondar comprehensive specialized hospitals in the Amhara Region, Northwest Ethiopia: A case-control study
Source: PLoS One. 2024 Jul 9;19(7):e0289450. doi: 10.1371/journal.pone.0289450 (PMC11232969; doi:10.1371/journal.pone.0289450)
Supplement: S2 Table — (DOCX) [file pone.0289450.s004.docx]

Table 2: Behavioral characteristics of patients on second-line ART at FHCSH and UGCSH; Amhara Region, Northwest Ethiopia from September to December 2021.

| General variables | Variables category | Frequency of Virological failure. No. (%) | | Total No. (%) | |
| --- | --- | --- | --- | --- | --- |
|  |  | Cases (N=59)  No. (%) | Controls (N=153)  No. (%) | 212 |  |
| Alcohol consumption | Yes | 9(15.3) | 26(17) | 35(16.5) |  |
|  | No | 50(84.7) | 127(83) | 177(83.5) |  |
| Using condom | Yes | 6(10.2) | 59(38.6) | 65(30.7) |  |
|  | No | 53(89.8) | 94(61.4) | 147(69.374.5) |  |
| Disclosure status | Disclosed | 13(22) | 87(56.9) | 100(47.2) |  |
|  | Not disclosed | 46(78) | 66(43.1) | 112(52.8) |  |
| Smoking cigarettes | Yes | 5(8.5) | 1(0.7) | 6(2.8) |  |
|  | No | 54(91.5) | 152(99.3) | 206(97.2) |  |
| Level of Adherence | Good | 13(22) | 91(59.5) | 104(49) |  |
|  | Medium | 13(22) | 39(25.5) | 36(17) |  |
|  | Poor | 33(55.9) | 3(2) | 72(34) |  |
