# Supplementary material for: Determinants of virological failure among HIV clients on second-line antiretroviral treatment at Felege-hiwot and University of Gondar comprehensive specialized hospitals in the Amhara Region, Northwest Ethiopia: A case-control study
Source: PLoS One. 2024 Jul 9;19(7):e0289450. doi: 10.1371/journal.pone.0289450 (PMC11232969; doi:10.1371/journal.pone.0289450)
Supplement: S3 Table — (DOCX) [file pone.0289450.s005.docx]

Table 3: Clinical and immunological characteristics of patients on second-line ART at FHCSH and UGCSH; Amhara Region, Northwest Ethiopia from September to December 2021.

| General variables | Variables category | Frequency of Virological failure. No. (%) | | Total No. (%)  N = 212 |
| --- | --- | --- | --- | --- |
|  |  | Cases (N=59)  No. (%) | Controls (N=153)  No. (%) |  |
| Body mass index (BMI) | <18.5 | 20(33.9) | 25(16.3) | 45(21.2) |
|  | 18.5 – 25 | 36(61) | 106(69.3) | 142(67) |
|  | >25 – 30 | 2(3.4) | 21(13.7) | 23(10.8) |
|  | >30 | 1(1.7) | 1(0.7) | 2(0.9) |
| CD4 count (cells/mm3) | <200 | 24(40.7) | 62(40.5) | 86(40.6) |
|  | 200 – 350 | 21(35.6) | 53(34.6) | 74(34.9) |
|  | 350.01 - 500 | 9(15.3) | 29(19) | 38(17.9) |
|  | >500 | 5(8.5) | 9(5.9) | 14(6.6) |
| Nutritional status | Normal | 33(55.9) | 104(68) | 137(64.6) |
|  | MAM (Moderately) | 10(16.9) | 25(16.3) | 35(16.5) |
|  | SAM (severely) | 8(13.6) | 7(4.6) | 15(7.1) |
|  | Overweight | 8(13.6) | 17(11.1) | 25(11.8) |
| Co-trimoxazole started | Yes | 28(47.5) | 74(48.4) | 102(48.1) |
|  | No | 31(52.5) | 79(51.6) | 110(51.9) |
| Fluconazole started | Yes | 3(5.1) | 22(14.4) | 25(11.8) |
|  | No | 56(94.9) | 131(85.6) | 187(88.2) |
| Viral load when switched to second line ART (copes/ml) | <150 | 18(30.5) | 104(68) | 122(57.5) |
|  | 150 – 999.999 | 11(18.6) | 8(5.2) | 19(9) |
|  | >1000 | 30(50.8) | 41(26.8) | 71(33.5) |
| WHO HIV clinical stage | stage I | 49(83.1) | 151(98.7) | 200(94.3) |
|  | stage II | 1(1.7) | 1(0.7) | 2(0.9) |
|  | stage III | 9(15.3) | 1(0.7) | 10(4.7) |
| Duration in first line ART /in years/ | <2 years | 6(10.2) | 26(17) | 32(15.1) |
|  | 2 -5 years | 16(27.1) | 58(37.9) | 74(34.9) |
|  | 5.01 – 10 years | 30(50.8) | 60(39.2) | 90(42.5) |
|  | > 10 years | 7(11.9) | 9(5.9) | 16(7.5) |
