# Supplementary material for: Determinants of virological failure among HIV clients on second-line antiretroviral treatment at Felege-hiwot and University of Gondar comprehensive specialized hospitals in the Amhara Region, Northwest Ethiopia: A case-control study
Source: PLoS One. 2024 Jul 9;19(7):e0289450. doi: 10.1371/journal.pone.0289450 (PMC11232969; doi:10.1371/journal.pone.0289450)
Supplement: S4 Table — (DOCX) [file pone.0289450.s006.docx]

Table 4: Factors associated to second line ART Virological failure among second-line HIV Patients at FHCSH and UGCSH; Amhara Region, Northwest Ethiopia from September first, 2021, to December last 2021.

| General variables | Variables category | Virological failure | | Odd Ratios | | | |
| --- | --- | --- | --- | --- | --- | --- | --- |
|  |  | Yes | No | COR (95% CL) | P- value | AOR (95% CL) | P-  value |
| Using condom | Yes | 6 | 59 | 1 |  |  |  |
|  | No | 53 | 94 | 5.54 (2.3-13.7) | 0.000 | 4.5(1.63 – 12) | 0.004 |
| Disclosure status | Disclosed | 13 | 87 | 1 |  |  |  |
|  | Not disclosed | 46 | 66 | 4.6(2.33–9.34) | 0.000 | 3.4(1.5 – 7.8) | 0.003 |
| Level of  Adherence | Good | 13 | 91 | 1 |  |  |  |
|  | Medium | 13 | 23 | 3.95(1.6 - 9.7) | 0.003 | 3.7(1.3 - 10.7) | 0.014 |
|  | Poor | 33 | 39 | 5.9(2.8 – 12.4) | 0.000 | 5.3(2.2 – 12.5) | 0.000 |
| Nutritional status | Normal | 33 | 104 | 1 |  |  |  |
|  | MAM | 10 | 25 | 1.26(0.55 – 3) | 0.58 | 1.2(0.4 – 3) | 0.75 |
|  | SAM | 8 | 7 | 3.6(1.2 – 10.7) | 0.021 | 3.56(1 – 14.4) | 0.075 |
|  | Overweight | 8 | 17 | 1.48(0.6 – 3.8) | 0.045 | 1.6 (0.4 – 5) | 0.46 |
| Viral load when switched to second line ART (copes/ml) | <150 | 18 | 104 | 1 |  |  |  |
|  | 150– <1000 | 11 | 8 | 7.9(2.8 – 22) | 0.000 | 5.4(1.5 – 19) | 0.009 |
|  | > 1000 | 30 | 41 | 4.2(2.1 – 8.4) | 0.000 | 3.56(1.5 - 8) | 0.002 |
