# Supplementary material for: Determinants of virological failure among HIV clients on second-line antiretroviral treatment at Felege-hiwot and University of Gondar comprehensive specialized hospitals in the Amhara Region, Northwest Ethiopia: A case-control study
Source: PLoS One. 2024 Jul 9;19(7):e0289450. doi: 10.1371/journal.pone.0289450 (PMC11232969; doi:10.1371/journal.pone.0289450)
Supplement: Attachment — Submitted filename: Comments.docx [file pone.0289450.s007.docx]

**Title modification:** Determinants of virological failure among HIV clients on second-line antiretroviral treatment at Felege-hiwot and University of Gondar comprehensive specialized hospitals Amhara region, Northwest Ethiopia: a case-control study

**Background:** it doesn’t show the gap that the research was required to be done. Further justification for the study will be required to be published. A lot of studies were done in Ethiopia in this title. How new findings come with this study?

**Methods:** Why patients on third line regimens included? Is your findings generalizable to such ART patients?

The study included ART patients on second line treatment for 6 months, but to detect virological failure at least two consecutive viral load results are required to be done after 6 months of treatment follow up. Based on this scenario, could this method follow a correct procedure? Please have in details your methodology and the real situation. So, your study should include those ART patients at least on second line treatment for 9 months.
