# Supplementary material for: Determinants of virological failure among HIV clients on second-line antiretroviral treatment at Felege-hiwot and University of Gondar comprehensive specialized hospitals in the Amhara Region, Northwest Ethiopia: A case-control study
Source: PLoS One. 2024 Jul 9;19(7):e0289450. doi: 10.1371/journal.pone.0289450 (PMC11232969; doi:10.1371/journal.pone.0289450)
Supplement: Attachment — Submitted filename: Rebutal ltter.docx [file pone.0289450.s008.docx]

Journal Submissions Rebuttal Letter, (Response to Reviewers)

Yoon-Seok Chung
Academic Editor
PLOS ONE

San Francisco, California, USA

2/11/2024

Dear Mr. Chung.

We are grateful for the opportunity to submit a revised draft of our manuscript entitled, " Determinants of virological failure among HIV clients on second-line antiretroviral treatment at Felege-hiwot and University of Gondar comprehensive specialized hospitals in the Amhara region, Northwest Ethiopia: a case-control study" to plos one. We also appreciate the time and effort you and each of the reviewers have dedicated to providing insightful feedback on ways to strengthen our paper. Thus, it is with great pleasure that we resubmit our article for further consideration. We have incorporated changes that reflect the detailed suggestions you have graciously provided. We also hope that our edits and the responses we provide below satisfactorily address all the issues and concerns you and the reviewers have noted.

To facilitate your review of our revisions, the following is a point-by-point response to the questions and comments delivered in your letter dated 2023, December 4.

**Editor suggestions:**

1. Please ensure that your manuscript meets PLOS ONE's style requirements, including those for file naming.

**Response:** Thank you for your suggestion; we have tried to amend our manuscript based on PLOS ONE style templates you recommended. And we also referred plos one submission guidelines, Which is available at <https://journals.plos.org/plosone/s/submission-guidelines>

2. We noticed you have some minor occurrence of overlapping text with the following previous publication(s), which needs to be addressed:

**Response:** we would like to say thank you for this and have incorporated this suggestion throughout our paper. However, we want to say that the overlapping occurs by chance and not copied intentionally. Scientifical terms like definitions, facts and guidelines may be used in different literature with the same expressions. Even the one mentioned in the first link, <https://www.researchgate.net/publication/361344207_Referral_challenges_and_outcomes_of_neonates_received_at_Muhimbili_National_Hospital_Dar_es_Salaam_Tanzania>. We did not use this paper as a reference.

3. We suggest you thoroughly copyedit your manuscript for language usage, spelling, and grammar

**Response:** In our revisions, we have attempted to copyedit our manuscript thoroughly for language usage, spelling, and grammar. Dr. Gebremariam Getaneh is assigned for this task. He had been working at Bahir Dar University, College of Medicine and Health science, School of Public Health for more than 12 years. now he is an assistant professor of Health Systems and Project Management at this university. He had good experience in research. I appreciate your recommendations to consider employing a professional scientific editing service. But, due to payment issues we unable to do that.

4. Please amend the manuscript submission data (via Edit Submission) to include author Gebremariam Getaneh.

**Response:** Thank you for providing these insights. We did it

5. Please amend your list of authors on the manuscript to ensure that each author is linked to an affiliation.

**Response:** I also thank you for this, we have made a correction on this part. Now, we think that each author is properly linked their affiliation. Since no author moved from their first workplace, there is no new affiliation list.

6. Please include your full ethics statement in the ‘Methods’ section of your manuscript file. In your statement, please include the full name of the IRB or ethics committee who approved or waived your study, as well as whether or not you obtained informed written or verbal consent. If consent was waived for your study, please include this information in your statement as well.

**Response:** Thank you for your suggestion. We have included it. We also attached the original ethical clearance paper obtained from Bahir Dar university institutional review board committee in the supporting information part.

7. Please include a copy of Table 7 which you refer to in your text.

**Response:** You have raised an important point; thank you for this. However, we only have 4 tables in the manuscript and there was no table 7. We wanted to say table 4, not table 7. We did a typing error here. We apologize for this, and we correct it as table 4 in our revised manuscript.

8. Please include captions for your Supporting Information files at the end of your manuscript, and update any in-text citations to match accordingly.

Response: We have included table captions, ethical clearance paper and annexes in our revised manuscript.

9. Please review your reference list to ensure that it is complete and correct. If you have cited papers that have been retracted, please include the rationale for doing so in the manuscript text or remove these references and replace them with relevant current references. Any changes to the reference list should be mentioned in the rebuttal letter that accompanies your revised manuscript.

**Response:** This is an interesting perspective. We have reviewed our references and made some adjustments. i.e., We remove duplicated list of references in the bibliography, we replace some references with another which has updated information. E.g. we replaced ‘’Avert; HIV AIDS’’ by WHO updated guideline and we also updated the information related with these references in our revision. We also made some modifications based on Vancouver reference style.

**Review Comments to the Author**

**Reviewer 1 comments:**

10. By case control able to identify associated factors but not identify predictors.

**Response:** That is an interesting query. even though there is a little difference between the two, predictors and associated factors, both indicates the characteristic of the patient's response (outcome) to a specified treatment or outcome of interest. That is why we used the word predictors. But by taking reviewer 1and reviewer 3 comments into consideration, we modify the tittle as ‘’ Determinants of virological failure among HIV clients on second-line antiretroviral treatment at Felege-hiwot and University of Gondar comprehensive specialized hospitals in the Amhara region, Northwest Ethiopia: a case-control study’’. Since determinants are variables causally related to the outcome of interest, we think determinant would be more appropriate for this tittle.

11. More detail information about socio-demographic characteristics of the study participants in the abstract, correct this based on your objectives.

**Response:** we would like to say thank You for the point you raised; however, out of the total of socio-demographic characteristics we only mention 3 that accounts a high percentage as compared to their counter parts. Even if we tried to amend this section by considering your suggestion.

12. Introduction, more than three pages – need to minimize to two pages

**Response:** This is an interesting perspective. We minimize this section nearly to two pages. While doing this, we tried to keep main points that the introduction should contain.

13. Methods and materials
Study settings
Bahir Dar city - Geographical coordinates of Bahir Dar city are 11° 36' 0" North, 37° 23' 0" East and an Elevation of 1,800 m (5,900 ft.) above sea level (52,53). The city of Gondar - It founds at 12° 36′ 0″ N, latitude 37° 28′ 0″ E longitude coordinates and an elevation of 2133 m above sea level. (54,55). All the above information - not link with the objectives of this research?

**Response:** We agree with you and have incorporated this suggestion. We removed these explanations in our revised manuscript.

13. Sample size determination and sampling techniques; More detail – focus on main concepts - minimize in order to fit the publication standard. Overall – the method section More detail – focus on main concepts - minimize in order to fit the publication standard.

**Response:** We agree with you and have incorporated this suggestion throughout our paper.

14. In bi-variable logistic regression from a total of 36 variables the following: Not using condom, Non-disclosure about HIV status, poor Level of Adherence, severely malnutrition nutritional status and Viral load >1000 copes/ml when switched to second line ART were significantly associated with second line virological ART failure (P-value<0.25). All the above variables are not significantly associated but selected for multivariable binary logistic regression analysis.

**Response:** Thank you for providing these insights. In this paragraph, we want to show the variables that were statistically significant in bi-variable logistic regression analysis and included in the multivariable binary logistic regression analysis. So, we accept your comments and made a correction.

**Reviewer 2 comments:**

15. Abstract section; Conclusion part: You don’t have to repeat what was mentioned in the result section. Explain it in other terms. The statement “…

**Response:** Thank you for your suggestion. We made an effort to explain it in other ways, However, we tried to explain the conclusion part best shows what was found in our investigations.

16. counseling about the importance of disclosure and good adherence for health care providers are crucial” not clear. Would you please re-phrase it in better terms?

**Response:** you have forwarded an important suggestion. The sentences ‘’counseling about the importance of disclosure and good adherence’’ It is an ambiguous sentence. Here we want to Say ‘’health care providers should give good counseling service regarding the importance of disclosure and good adherence to patients on ART’’. but our investigations did not show counseling activities in the study sites, and we think recommendation about counseling in this paper will not be appropriate. Due to this reason, we remove this sentence in our revised manuscript.

17. Introduction; The detailed description of prior 1st line ART regimen (NNRTI-based regimen) was not required. Focus on magnitude, and details on factors associated with 2nd line ART regimen failure at international, continental, and national level.

**Response:** We agree with you and have incorporated this suggestion throughout our paper. And we tried to summaries the factors associated with 2nd line ART regimen failure found from different literatures.

Acronym use like ‘VF’, I think for virologic failure, should be mentioned after what is.

**Response:** thank you for this suggestion, we corrected it.

18. Methods; Population subsection
a. Describe source and study population separately

**Response:** we described separately based on your comments,
b. Definition of terms, Relocate ‘definition of terms’ at the end of methods section.

**Response:** We agreed with your assessment and relocated it at the end of methods section.

c. Data processing and analysis, Line-3: …. was employed to identify determinates of second line ART virological failure’. Do you mean determinants…?

**Response:** Yes; thank you for this. We correct it as determinants in our revision.
Line-5: …. multivariate logistic regression mode and…. Do you mean model?

**Response:** Yes; also thank you for this. We correct it as model in our revision.

19. The variable, Age (in yrs), is not properly grouped. Stratify age interval according to the standard.

**Response:** There have been many ways to categorize the ages of individuals. Age grouping can be done with different ranges according to the type of study, the objective of investigation and the nature of participants. However, the Appropriate classifications of the age group for risk factor assessment recommend by different research are 0-14 years old (children group), **15-24 years (Youth group), 25-64 years (Adults group) and 65 years and over (**elderly **group).** we believe that this age grouping would be more appropriate for our investigation.

20. Foot note for MAM and SAM under the table-3 to explain what it is

**Response:** Thank you for your suggestion. We explained it in our revision.

21. Regarding variables, BMI and Nutritional status: BMI indicate nutritional status. Why a need another variable as ‘nutritional status’

**Response:** You have raised an important question. nutritional status can be measured by different anthropometric indicators. Commonly BMI and Mid Upper Arm Circumference (MUAC). MUAC is the only anthropometric measure for assessing nutritional status among pregnant women and target children for severe acute malnutrition (SAM) and moderate acute malnutrition (MAM). Body mass index is the best measure of non-pregnant adult nutritional status. So, we planned at the bigging if we get pregnant women and target children, and we believed that collecting the two information would be more appropriate and will not be disadvantage.

22. There is no any description in the manuscript regarding switching to 2nd line ART regimen other than virologic failure i.e viral load>1000 copies/ml. Describe reason for switching other than VF for those with VL<1000 copies/ml. Clinical and immunological characteristics

**Response:** You have raised an important point; however, our study aimed at identifying factors for virological failures only. This makes our investigation is somewhat different from previous studies. In settings whenever viral load monitoring is possible, using clinical and immunological characteristics of the patient to rule out ART failure is not recommended ‘’ updated 2018 WHO ART guideline’’. In our study area, routine and high viral load monitoring activity has been done. Thus, we believe that [Clinical and immunological characteristics] would be outside the scope of our paper.

23. Don’t start a sentence with a number. It should be with a text.

**Response:** we thank for this; we had made a correction.

24.Insert a column for p-value for COR and AOR. Your p-value to test significance was at 0.05. Why a need to mention at p-value of 0.01, 0.005, 0.001. Testing at p-value at 0.05 is adequate.

**Response:** We have taken your comments into consideration and thank you for them.

25. Under the title “Factors associated to second line ART virological failure”, there were 36 variables tested for bivariate analysis. You need to mention those variables tested but not significant in bivariate analysis.

**Response:** You have asked an interesting question. In our study, we assessed 36 variables using our data collection tools. Unfortunately, some variables were not found. For example, we assessed ‘’TB- comorbidity’’ in our investigation, but we did not find any TB-comorbidity case. In this sentence we want to indicate the number of variables assessed during data collection. We apologize for the expression '36 variables tested for bivariate analysis'. The number of variables tested were those listed in table1, table 2 and table 3. we hope that the edited section of our manuscript will clarifies this expression better.

26. The statement: “Moreover, the likely hood of developing second line virologic failure among patients with Viral load >1000 copes/ml when switched to second line ART medication were 3.5 times (AOR=3.56, 95% CI: 1.5 - 8) more likely as compared to those patients who had Viral load result <1000 copes/ml”. Correction should be made in abstract, results and discussion section.It should be re-written as “…those with VL>1000 copies/ml had 3.5 times more to develop VF as compared to those with VL<150 copies /ml…” and ““…those with VL>150-999 copies/ml had 5 times more to develop VF as compared to those with VL<150 copies /ml…”

**Response:** We agree with your assessment and have incorporated this suggestion throughout our revised manuscript.

27. Discussion; Citation of reference should be at the end of a sentence, not in the middle of a sentence.

**Response:** Thank you for providing these insights. We have made a correction.

28. Acronym ‘…meds…’ do you mean medications. Correct it as required.

**Response:** Yes, it was to mean medications. We corrected it.

29. Revise a statement “Double-edged sword of disclosure on ART adherence…”
You mentioned the advantage of disclosure on ART adherence, what was the disadvantage of disclosure on ART adherence.

**Response:** great look you did, thank you for this. We have elaborated the disadvantage of disclosure on ART adherence throughout our discussion in our revised manuscript.

30. Reference; It was referenced manually. It should be referenced using Mandalay or EndNote electronically.

**Response:** we agree. In our revision, we used Mendeley Reference Manager.

**Reviewer 3 comments:**

31. A language editing service is required for the paper to be published. A lot of findings are there regarding this topic, so the findings may not add new things to the literature communities.

**Response:** In our revisions, we have attempted to copyedit our manuscript thoroughly for language usage, spelling, and grammar. Dr. Gebremariam Getaneh who is an assistant professor of Health Systems and Project Management at Bahir Dar University devoted his time for this. He had a good experience on this. We believe that this issue would be addressed better in our revision.

32. A lot of findings are there regarding this topic, so the findings may not add new things to the literature communities.
**Response:** There are multiple reasons that our study makes a significant contribution to the literature. 1. Different research has showed that, data available about the risk factors for second-line ART virological failure in sub-Sahara Africa is limited. Most of the previous studies conducted were focused on first line ART failure. Even though some investigations were done on this area, the majority were used clinical and immunological characteristics of the patient, but it is not recommended by WHO guideline whenever viral load monitoring is possible.2. The problem becomes increasing from time to time. Now, it is one of a public health issue worldwide. 3. No investigations were done regarding the risk factors of second-line ART virological failure in the current study area. 4. This finding will use as a source of information for systematic reviews. 5. It is also useful for stakeholders to set programs and action plans to reduce second-line ART drug resistance.

33. Title modification is needed.

**Response:** we accepted your comment and modified the tittle.

34. Background: It doesn’t show the gap that the research was required to fill. Further justification for the study will be required to be published. A lot of studies were done in Ethiopia for this title. How do new findings come with this study?
**Response**: Your comment has taken into consideration, and we have tried to justify the reason why this investigation is required. We also elaborated this idea above. (Response for que. 32.)

35. Methods: Why are patients on third-line regimens included? Are your findings generalizable to such ART patients? The study included ART patients on second-line treatment for 6 months, but to detect virological failure, at least two consecutive viral load results are required after 6 months of treatment follow-up. Based on this scenario, could this method follow the correct procedure? Please provide in detail your methodology and the real situation. So, your study should include those ART patients, at least on second-line treatment for 9 months.

**Response:** during our data collection, we collect data only from patients who were on second-line ART. But some patients were transferred to third-line ART during data collection following to second-line ART failure. That is why we included patients on third-line regimen. But in our revision, we have made a correction (delete the word third line) throughout our paper to make it clearer and avoid confusion.

In our research proposal, we planned to include ART patients on second-line treatment who had taken second-line ART drugs for at least 6 months at the start of data collection. Then the first viral load test will be done. Following this, the second viral load test will be done after 3 months Since our study period is 4 months (from September to December), the patient will have two consecutive viral load results. Unfortunately, we found that all patients who were on second-line ART had at least one viral load test result during data collection. i.e. all study participants were on second-line ART treatment for more than 9 months.

This scenario was well stated in the abstract part method and material section (Patients who had two viral load results ≥1000 copies/ml within a 3-month interval after taking ART drugs for at least 6 months were cases whereas ≤1,000 copies/ mL was controls). It is also clearly explained in the methodology part **sampling techniques, Case, and control selection. Saying this,** we have made some modifications in the population section to make it clearer and avoid confusion.
