# Supplementary material for: Determinants of virological failure among HIV clients on second-line antiretroviral treatment at Felege-hiwot and University of Gondar comprehensive specialized hospitals in the Amhara Region, Northwest Ethiopia: A case-control study
Source: PLoS One. 2024 Jul 9;19(7):e0289450. doi: 10.1371/journal.pone.0289450 (PMC11232969; doi:10.1371/journal.pone.0289450)
Supplement: Peer review history

# Peer review: Decision Letter 0

**DOI:** 10.1371/journal.pone.0289450.r001

**Authors:** Yoon-Seok Chung

14 Dec 2023

PONE-D-23-20506Predictors of second-line antiretroviral treatment virological failure at Felege hiwot and  University of Gondar comprehensive specialized hospitals Amhara region, Northwest Ethiopia: a case-control studyPLOS ONE

Dear Dr. Wobetu,

Thank you for submitting your manuscript to PLOS ONE. After careful consideration, we feel that it has merit but does not fully meet PLOS ONE’s publication criteria as it currently stands. Therefore, we invite you to submit a revised version of the manuscript that addresses the points raised during the review process.

Please submit your revised manuscript by Jan 28 2024 11:59PM. If you will need more time than this to complete your revisions, please reply to this message or contact the journal office at plosone@plos.org. When you're ready to submit your revision, log on to https://www.editorialmanager.com/pone/ and select the 'Submissions Needing Revision' folder to locate your manuscript file.

Please include the following items when submitting your revised manuscript:

- A rebuttal letter that responds to each point raised by the academic editor and reviewer(s). You should upload this letter as a separate file labeled 'Response to Reviewers'.
- A marked-up copy of your manuscript that highlights changes made to the original version. You should upload this as a separate file labeled 'Revised Manuscript with Track Changes'.
- An unmarked version of your revised paper without tracked changes. You should upload this as a separate file labeled 'Manuscript'.

If you would like to make changes to your financial disclosure, please include your updated statement in your cover letter. Guidelines for resubmitting your figure files are available below the reviewer comments at the end of this letter.

If applicable, we recommend that you deposit your laboratory protocols in protocols.io to enhance the reproducibility of your results. Protocols.io assigns your protocol its own identifier (DOI) so that it can be cited independently in the future. For instructions see: https://journals.plos.org/plosone/s/submission-guidelines#loc-laboratory-protocols. Additionally, PLOS ONE offers an option for publishing peer-reviewed Lab Protocol articles, which describe protocols hosted on protocols.io. Read more information on sharing protocols at https://plos.org/protocols?utm_medium=editorial-email&utm_source=authorletters&utm_campaign=protocols.

We look forward to receiving your revised manuscript.

Kind regards,

Yoon-Seok Chung

Academic Editor

PLOS ONE

Journal requirements:

1. When submitting your revision, we need you to address these additional requirements.

Please ensure that your manuscript meets PLOS ONE's style requirements, including those for file naming. The PLOS ONE style templates can be found at 

https://journals.plos.org/plosone/s/file?id=wjVg/PLOSOne_formatting_sample_main_body.pdf and 

https://journals.plos.org/plosone/s/file?id=ba62/PLOSOne_formatting_sample_title_authors_affiliations.pdf.

2. We noticed you have some minor occurrence of overlapping text with the following previous publication(s), which needs to be addressed:

https://www.researchgate.net/publication/361344207_Referral_challenges_and_outcomes_of_neonates_received_at_Muhimbili_National_Hospital_Dar_es_Salaam_Tanzania

https://bmcinfectdis.biomedcentral.com/articles/10.1186/s12879-019-4148-3

file:///home/nkw-la22-034/Downloads/WHO-CDS-HIV-19.21-eng.pdf

https://www.poz.com/basics/hiv-basics/hiv-drug-resistance?utm_campaign=301_Redirect&utm_source=aidsmeds

In your revision ensure you cite all your sources (including your own works), and quote or rephrase any duplicated text outside the methods section. Further consideration is dependent on these concerns being addressed.

3. We suggest you thoroughly copyedit your manuscript for language usage, spelling, and grammar. If you do not know anyone who can help you do this, you may wish to consider employing a professional scientific editing service. 

Whilst you may use any professional scientific editing service of your choice, PLOS has partnered with both American Journal Experts (AJE) and Editage to provide discounted services to PLOS authors. Both organizations have experience helping authors meet PLOS guidelines and can provide language editing, translation, manuscript formatting, and figure formatting to ensure your manuscript meets our submission guidelines. To take advantage of our partnership with AJE, visit the AJE website (http://learn.aje.com/plos/) for a 15% discount off AJE services. To take advantage of our partnership with Editage, visit the Editage website (www.editage.com) and enter referral code PLOSEDIT for a 15% discount off Editage services.  If the PLOS editorial team finds any language issues in text that either AJE or Editage has edited, the service provider will re-edit the text for free.

Upon resubmission, please provide the following:

The name of the colleague or the details of the professional service that edited your manuscript

A copy of your manuscript showing your changes by either highlighting them or using track changes (uploaded as a *supporting information* file)

A clean copy of the edited manuscript (uploaded as the new *manuscript* file)".

4. Please amend the manuscript submission data (via Edit Submission) to include author Gebremariam Getaneh.

5. Please amend your list of authors on the manuscript to ensure that each author is linked to an affiliation. Authors’ affiliations should reflect the institution where the work was done (if authors moved subsequently, you can also list the new affiliation stating “current affiliation:….” as necessary).

6. Please include your full ethics statement in the ‘Methods’ section of your manuscript file. In your statement, please include the full name of the IRB or ethics committee who approved or waived your study, as well as whether or not you obtained informed written or verbal consent. If consent was waived for your study, please include this information in your statement as well. 

7. Please include a copy of Table 7 which you refer to in your text.

8. Please include captions for your Supporting Information files at the end of your manuscript, and update any in-text citations to match accordingly. Please see our Supporting Information guidelines for more information: http://journals.plos.org/plosone/s/supporting-information. 

9. Please review your reference list to ensure that it is complete and correct. If you have cited papers that have been retracted, please include the rationale for doing so in the manuscript text, or remove these references and replace them with relevant current references. Any changes to the reference list should be mentioned in the rebuttal letter that accompanies your revised manuscript. If you need to cite a retracted article, indicate the article’s retracted status in the References list and also include a citation and full reference for the retraction notice.

[Note: HTML markup is below. Please do not edit.]

Reviewers' comments:

Reviewer's Responses to Questions

**Comments to the Author**

1. Is the manuscript technically sound, and do the data support the conclusions?

The manuscript must describe a technically sound piece of scientific research with data that supports the conclusions. Experiments must have been conducted rigorously, with appropriate controls, replication, and sample sizes. The conclusions must be drawn appropriately based on the data presented. 

Reviewer #1: Yes

Reviewer #2: Yes

Reviewer #3: Partly

**********

2. Has the statistical analysis been performed appropriately and rigorously? 

Reviewer #1: Yes

Reviewer #2: Yes

Reviewer #3: Yes

**********

3. Have the authors made all data underlying the findings in their manuscript fully available?

The PLOS Data policy requires authors to make all data underlying the findings described in their manuscript fully available without restriction, with rare exception (please refer to the Data Availability Statement in the manuscript PDF file). The data should be provided as part of the manuscript or its supporting information, or deposited to a public repository. For example, in addition to summary statistics, the data points behind means, medians and variance measures should be available. If there are restrictions on publicly sharing data—e.g. participant privacy or use of data from a third party—those must be specified.

Reviewer #1: Yes

Reviewer #2: Yes

Reviewer #3: Yes

**********

4. Is the manuscript presented in an intelligible fashion and written in standard English?

PLOS ONE does not copyedit accepted manuscripts, so the language in submitted articles must be clear, correct, and unambiguous. Any typographical or grammatical errors should be corrected at revision, so please note any specific errors here.

Reviewer #1: No

Reviewer #2: Yes

Reviewer #3: No

**********

5. Review Comments to the Author

Please use the space provided to explain your answers to the questions above. You may also include additional comments for the author, including concerns about dual publication, research ethics, or publication ethics. (Please upload your review as an attachment if it exceeds 20,000 characters)

Reviewer #1: In the abstract

Objective: To assess the predictors of second line Anti-Retroviral Treatment virological failure among second line ART users

Method and materials: Institutional based unmatched case control study design was conducted.

By case control able to identify associated factors but not identify predictors.

Results: Out of 216 patients recruited, 212 were participated with a response rate of 98.2%. Among the participants, 117 (55.2%) were males and 187 (88.2%) were urban dwellers. 208 (98.1%) of the respondents had age > 24 years, 73 (34.4%) had elementary level of education, 72(34%) had poor ART adherence and 112(52.8) did not disclose their HIV status.

More detail information about socio-demographic characteristics of the study participants – correct this based on your objectives.

Introduction

More than three pages – need to minimize to two pages

Methods and materials

Study settings

Bahir Dar city - Geographical coordinates of Bahir Dar city are 11° 36' 0" North, 37° 23' 0" East and an Elevation of 1,800 m (5,900 ft.) above sea level (52,53).

The city of Gondar - It founds at 12° 36′ 0″ N, latitude 37° 28′ 0″ E longitude coordinates and an elevation of 2133 m above sea level. (54,55).

All the above information - not link with the objectives of this research?

Sample size determination and sampling techniques

Sample size was calculated using EPIINFO version 7.2.4 (a two-population proportion formula). n= sample size (number of participants), 95% confidence level) Z α/2=1.96 (for 0.05 significance level), for 80% power, Z�= 0.84, r = 1/3 (the ratio of cases to controls), p1= 31% (% of cases among exposed), p2= 11.2% (% of cases among non-exposed). From previous study conducted in Wollo, Amhara Regional State, Northeast Ethiopia, the following significant predictors for second-line antiretroviral treatment virological failure were found, Age 15 – 29 years, Poor adherence, Have no disclosure status, BMI < 16 k.g/m2, CD4 count < 100cells/mm3 , CD4 count 100 – 350 cells/mm3, Having opportunistic infections (51). We used this report as a base line data, the minimum sample size including 10% non-respondent rate for this study was 206 participants (52 cases and 154 controls). But we recruited 216 patients (all the cases 60) and 156 controls.

Sampling techniques

Case selection: All HIV patients who had taken a second line ART for at least six months and had developed a virological failure were selected and listed from the patients registration book at FHCSH and UGCSH during the study period. There were 31 patients at FHCSH and 29 patients at UGCSH who were failed for second line ART. Control selection: All HIV patients who had taken…

More detail – focus on main concepts - minimize in order to fit the publication standard

Over all – the method section More detail – focus on main concepts - minimize in order to fit the publication standard

Result

Factors associated to second line ART virological failure

In bi-variable logistic regression from a total of 36 variables the following: Not using condom, Non-disclosure about HIV status, poor Level of Adherence, severely malnutrition nutritional status and Viral load >1000 copes/ml when switched to second line ART were significantly associated with second line virological ART failure (P-value<0.25).

All the above variables are not significantly associated but selected for multivariable binary logistic regression analysis

Reviewer #2: Comments to the authors

1. Abstract section

Conclusion part: You don’t have to repeat what was mentioned in the result section. Explain it in other terms. The statement “…counseling about the importance of disclosure and good adherence for health care providers are crucial” not clear. Would you please re-phrase it in better terms?

2. Introduction

. The detailed description of prior 1st line ART regimen (NNRTI-based regimen) was not required. Focus on magnitude, and details on factors associated with 2nd line ART regimen failure at international, continental, and national level.

. Acronym use like ‘VF’ , I think for virologic failure, should be mentioned after what is.

3. Methods

a. Population subsection

. Describe source and study population separately

b. Definition of terms

Relocate ‘definition of terms’ at the end of methods section.

c. Data processing and analysis

. Line-3: ….was employed to identify determinates of second line ART virological failure’. Do you mean determinants…?

Line-5: ….multivariate logistic regression mode and…. Do you mean model?

4. Results

a. Table-1

. The variable, Age (in yrs), is not properly grouped. Stratify age interval according to the standard.

b. Table-3

. Foot note for MAM and SAM under the table-3 to explain what it is

. Regarding variables, BMI and Nutritional status: BMI indicate nutritional status. Why a need another variable as ‘nutritional status’. Severity of undernutrition can be verified based on BMI like mild undernutrition as BMI=17.5-18.5kg/m2, Moderate undernutrition as 16-17.5kg/m2, and severe undernutrition as <16kg/m2

. The variable “Viral load when switched to 2nd line ART”

There is no any description in the manuscript regarding switching to 2nd line ART regimen other than virologic failure i.e viral load>1000 copies/ml. Describe reason for switching other than VF for those with VL<1000 copies/ml.

. Clinical and immunological characteristics

Don’t start a sentence with a number. It should be with a text

Eg. “8(13.6%) of the cases and 7(4.6%) of the controls…” It should be “Eight (13.6%) of the cases…”

Table-4

. Insert a column for p-value for COR and AOR

. Your p-value to test significance was at 0.05. Why a need to mention at p-value of 0.01, 0.005, 0.001. Testing at p-value at 0.05 is adequate.

Under the title “Factors associated to second line ART virological failure”, there were 36 variables tested for bivariate analysis. You need to mention those variables tested but not significant in bivariate analysis.

The statement:

“Moreover, the likely hood of developing second line virologic failure among patients with Viral

load >1000 copes/ml when switched to second line ART medication were 3.5 times (AOR=3.56,

95% CI: 1.5 - 8) more likely as compared to those patients who had Viral load result <1000

copes/ml”.

Correction should be made in abstract, results and discussion section.

It should be re-written as “…those with VL>1000 copies/ml had 3.5 times more to develop VF as compared to those with VL<150 copies /ml…”

and ““…those with VL>150-999 copies/ml had 5 times more to develop VF as compared to those with VL<150 copies /ml…”

Discussion

. Citation of reference should be at the end of a sentence, not in the middle of a sentence.

. Acronym ‘…meds…’ do you mean medications. Correct it as required.

. Revise a statement “Double-edged sword of disclosure on ART adherence…”

You mentioned the advantage of disclosure on ART adherence, what was the disadvantage of disclosure on ART adherence.

Reference

It was referenced manually. It should be referenced using Mandalay or EndNote electronically.

Reviewer #3: A language editing service is required for the paper to be published. A lot of findings are there regarding this topic, so the findings may not add new things to the literature communities.

Title modification: Determinants of virological failure among HIV clients on second-line antiretroviral treatment at Felege-hiwot and University of Gondar comprehensive specialized hospitals in the Amhara region, Northwest Ethiopia: a case-control study

Background: It doesn’t show the gap that the research was required to fill. Further justification for the study will be required to be published. A lot of studies were done in Ethiopia for this title. How do new findings come with this study?

Methods: Why are patients on third-line regimens included? Are your findings generalizable to such ART patients?

The study included ART patients on second-line treatment for 6 months, but to detect virological failure, at least two consecutive viral load results are required after 6 months of treatment follow-up. Based on this scenario, could this method follow the correct procedure? Please provide in detail your methodology and the real situation. So, your study should include those ART patients, at least on second-line treatment for 9 months.

**********

6. PLOS authors have the option to publish the peer review history of their article (what does this mean?). If published, this will include your full peer review and any attached files.

If you choose “no”, your identity will remain anonymous but your review may still be made public.

**Do you want your identity to be public for this peer review?** For information about this choice, including consent withdrawal, please see our Privacy Policy.

Reviewer #1: **Yes: **Esubalew Tesfahun (Ph.D)

Reviewer #2: **Yes: **Abilo Tadesse, MD

Reviewer #3: No

**********

[NOTE: If reviewer comments were submitted as an attachment file, they will be attached to this email and accessible via the submission site. Please log into your account, locate the manuscript record, and check for the action link "View Attachments". If this link does not appear, there are no attachment files.]

While revising your submission, please upload your figure files to the Preflight Analysis and Conversion Engine (PACE) digital diagnostic tool, https://pacev2.apexcovantage.com/. PACE helps ensure that figures meet PLOS requirements. To use PACE, you must first register as a user. Registration is free. Then, login and navigate to the UPLOAD tab, where you will find detailed instructions on how to use the tool. If you encounter any issues or have any questions when using PACE, please email PLOS at figures@plos.org. Please note that Supporting Information files do not need this step.

Attachment Submitted filename: Comments.docx

# Author comment: Author response to Decision Letter 0

**DOI:** 10.1371/journal.pone.0289450.r002

11 Feb 2024

Yoon-Seok Chung

Academic Editor

PLOS ONE

San Francisco, California, USA

2/11/2024

Dear Mr. Chung. 

We are grateful for the opportunity to submit a revised draft of our manuscript entitled, " Determinants of virological failure among HIV clients on second-line antiretroviral treatment at Felege-hiwot and University of Gondar comprehensive specialized hospitals in the Amhara region, Northwest Ethiopia: a case-control study" to plos one. We also appreciate the time and effort you and each of the reviewers have dedicated to providing insightful feedback on ways to strengthen our paper. Thus, it is with great pleasure that we resubmit our article for further consideration. We have incorporated changes that reflect the detailed suggestions you have graciously provided. We also hope that our edits and the responses we provide below satisfactorily address all the issues and concerns you and the reviewers have noted.

To facilitate your review of our revisions, the following is a point-by-point response to the questions and comments delivered in your letter dated 2023, December 4.

Editor suggestions:

1. Please ensure that your manuscript meets PLOS ONE's style requirements, including those for file naming.

Response: Thank you for your suggestion; we have tried to amend our manuscript based on PLOS ONE style templates you recommended. And we also referred plos one submission guidelines, Which is available at https://journals.plos.org/plosone/s/submission-guidelines

2. We noticed you have some minor occurrence of overlapping text with the following previous publication(s), which needs to be addressed:

Response: we would like to say thank you for this and have incorporated this suggestion throughout our paper. However, we want to say that the overlapping occurs by chance and not copied intentionally. Scientifical terms like definitions, facts and guidelines may be used in different literature with the same expressions. Even the one mentioned in the first link, https://www.researchgate.net/publication/361344207_Referral_challenges_and_outcomes_of_neonates_received_at_Muhimbili_National_Hospital_Dar_es_Salaam_Tanzania. We did not use this paper as a reference. 

3. We suggest you thoroughly copyedit your manuscript for language usage, spelling, and grammar

 Response: In our revisions, we have attempted to copyedit our manuscript thoroughly for language usage, spelling, and grammar. Dr. Gebremariam Getaneh is assigned for this task. He had been working at Bahir Dar University, College of Medicine and Health science, School of Public Health for more than 12 years. now he is an assistant professor of Health Systems and Project Management at this university. He had good experience in research. I appreciate your recommendations to consider employing a professional scientific editing service. But, due to payment issues we unable to do that. 

4. Please amend the manuscript submission data (via Edit Submission) to include author Gebremariam Getaneh.

 Response: Thank you for providing these insights. We did it 

5. Please amend your list of authors on the manuscript to ensure that each author is linked to an affiliation. 

Response: I also thank you for this, we have made a correction on this part. Now, we think that each author is properly linked their affiliation. Since no author moved from their first workplace, there is no new affiliation list.

6. Please include your full ethics statement in the ‘Methods’ section of your manuscript file. In your statement, please include the full name of the IRB or ethics committee who approved or waived your study, as well as whether or not you obtained informed written or verbal consent. If consent was waived for your study, please include this information in your statement as well. 

Response: Thank you for your suggestion. We have included it. We also attached the original ethical clearance paper obtained from Bahir Dar university institutional review board committee in the supporting information part. 

7. Please include a copy of Table 7 which you refer to in your text.

Response: You have raised an important point; thank you for this. However, we only have 4 tables in the manuscript and there was no table 7. We wanted to say table 4, not table 7. We did a typing error here. We apologize for this, and we correct it as table 4 in our revised manuscript. 

8. Please include captions for your Supporting Information files at the end of your manuscript, and update any in-text citations to match accordingly. 

Response: We have included table captions, ethical clearance paper and annexes in our revised manuscript.

9. Please review your reference list to ensure that it is complete and correct. If you have cited papers that have been retracted, please include the rationale for doing so in the manuscript text or remove these references and replace them with relevant current references. Any changes to the reference list should be mentioned in the rebuttal letter that accompanies your revised manuscript. 

Response: This is an interesting perspective. We have reviewed our references and made some adjustments. i.e., We remove duplicated list of references in the bibliography, we replace some references with another which has updated information. E.g. we replaced ‘’Avert; HIV AIDS’’ by WHO updated guideline and we also updated the information related with these references in our revision. We also made some modifications based on Vancouver reference style. 

 Review Comments to the Author

Reviewer 1 comments:

10. By case control able to identify associated factors but not identify predictors.

Response: That is an interesting query. even though there is a little difference between the two, predictors and associated factors, both indicates the characteristic of the patient's response (outcome) to a specified treatment or outcome of interest. That is why we used the word predictors. But by taking reviewer 1and reviewer 3 comments into consideration, we modify the tittle as ‘’ Determinants of virological failure among HIV clients on second-line antiretroviral treatment at Felege-hiwot and University of Gondar comprehensive specialized hospitals in the Amhara region, Northwest Ethiopia: a case-control study’’. Since determinants are variables causally related to the outcome of interest, we think determinant would be more appropriate for this tittle. 

 11. More detail information about socio-demographic characteristics of the study participants in the abstract, correct this based on your objectives.

Response: we would like to say thank You for the point you raised; however, out of the total of socio-demographic characteristics we only mention 3 that accounts a high percentage as compared to their counter parts. Even if we tried to amend this section by considering your suggestion.

12. Introduction, more than three pages – need to minimize to two pages

Response: This is an interesting perspective. We minimize this section nearly to two pages. While doing this, we tried to keep main points that the introduction should contain. 

13. Methods and materials

Study settings

Bahir Dar city - Geographical coordinates of Bahir Dar city are 11° 36' 0" North, 37° 23' 0" East and an Elevation of 1,800 m (5,900 ft.) above sea level (52,53). The city of Gondar - It founds at 12° 36′ 0″ N, latitude 37° 28′ 0″ E longitude coordinates and an elevation of 2133 m above sea level. (54,55). All the above information - not link with the objectives of this research?

Response: We agree with you and have incorporated this suggestion. We removed these explanations in our revised manuscript.

13. Sample size determination and sampling techniques; More detail – focus on main concepts - minimize in order to fit the publication standard. Overall – the method section More detail – focus on main concepts - minimize in order to fit the publication standard.

Response: We agree with you and have incorporated this suggestion throughout our paper.

14. In bi-variable logistic regression from a total of 36 variables the following: Not using condom, Non-disclosure about HIV status, poor Level of Adherence, severely malnutrition nutritional status and Viral load >1000 copes/ml when switched to second line ART were significantly associated with second line virological ART failure (P-value<0.25). All the above variables are not significantly associated but selected for multivariable binary logistic regression analysis.

Response: Thank you for providing these insights. In this paragraph, we want to show the variables that were statistically significant in bi-variable logistic regression analysis and included in the multivariable binary logistic regression analysis. So, we accept your comments and made a correction. 

Reviewer 2 comments:

15. Abstract section; Conclusion part: You don’t have to repeat what was mentioned in the result section. Explain it in other terms. The statement “…

Response: Thank you for your suggestion. We made an effort to explain it in other ways, However, we tried to explain the conclusion part best shows what was found in our investigations. 

16. counseling about the importance of disclosure and good adherence for health care providers are crucial” not clear. Would you please re-phrase it in better terms?

Response: you have forwarded an important suggestion. The sentences ‘’counseling about the importance of disclosure and good adherence’’ It is an ambiguous sentence. Here we want to Say ‘’health care providers should give good counseling service regarding the importance of disclosure and good adherence to patients on ART’’. but our investigations did not show counseling activities in the study sites, and we think recommendation about counseling in this paper will not be appropriate. Due to this reason, we remove this sentence in our revised manuscript.

17. Introduction; The detailed description of prior 1st line ART regimen (NNRTI-based regimen) was not required. Focus on magnitude, and details on factors associated with 2nd line ART regimen failure at international, continental, and national level.

Response: We agree with you and have incorporated this suggestion throughout our paper. And we tried to summaries the factors associated with 2nd line ART regimen failure found from different literatures.

Acronym use like ‘VF’, I think for virologic failure, should be mentioned after what is.

Response: thank you for this suggestion, we corrected it.

18. Methods; Population subsection

a. Describe source and study population separately

Response: we described separately based on your comments,

b. Definition of terms, Relocate ‘definition of terms’ at the end of methods section.

Response: We agreed with your assessment and relocated it at the end of methods section.

c. Data processing and analysis, Line-3: …. was employed to identify determinates of second line ART virological failure’. Do you mean determinants…?

Response: Yes; thank you for this. We correct it as determinants in our revision. 

Line-5: …. multivariate logistic regression mode and…. Do you mean model? 

Response: Yes; also thank you for this. We correct it as model in our revision.

19. The variable, Age (in yrs), is not properly grouped. Stratify age interval according to the standard.

Response: There have been many ways to categorize the ages of individuals. Age grouping can be done with different ranges according to the type of study, the objective of investigation and the nature of participants. However, the Appropriate classifications of the age group for risk factor assessment recommend by different research are 0-14 years old (children group), 15-24 years (Youth group), 25-64 years (Adults group) and 65 years and over (elderly group). we believe that this age grouping would be more appropriate for our investigation. 

20. Foot note for MAM and SAM under the table-3 to explain what it is

Response: Thank you for your suggestion. We explained it in our revision. 

21. Regarding variables, BMI and Nutritional status: BMI indicate nutritional status. Why a need another variable as ‘nutritional status’

Response: You have raised an important question. nutritional status can be measured by different anthropometric indicators. Commonly BMI and Mid Upper Arm Circumference (MUAC). MUAC is the only anthropometric measure for assessing nutritional status among pregnant women and target children for severe acute malnutrition (SAM) and moderate acute malnutrition (MAM). Body mass index is the best measure of non-pregnant adult nutritional status. So, we planned at the bigging if we get pregnant women and target children, and we believed that collecting the two information would be more appropriate and will not be disadvantage. 

22. There is no any description in the manuscript regarding switching to 2nd line ART regimen other than virologic failure i.e viral load>1000 copies/ml. Describe reason for switching other than VF for those with VL<1000 copies/ml. Clinical and immunological characteristics

Response: You have raised an important point; however, our study aimed at identifying factors for virological failures only. This makes our investigation is somewhat different from previous studies. In settings whenever viral load monitoring is possible, using clinical and immunological characteristics of the patient to rule out ART failure is not recommended ‘’ updated 2018 WHO ART guideline’’. In our study area, routine and high viral load monitoring activity has been done. Thus, we believe that [Clinical and immunological characteristics] would be outside the scope of our paper.

23. Don’t start a sentence with a number. It should be with a text. 

Response: we thank for this; we had made a correction.

24.Insert a column for p-value for COR and AOR. Your p-value to test significance was at 0.05. Why a need to mention at p-value of 0.01, 0.005, 0.001. Testing at p-value at 0.05 is adequate.

Response: We have taken your comments into consideration and thank you for them.

25. Under the title “Factors associated to second line ART virological failure”, there were 36 variables tested for bivariate analysis. You need to mention those variables tested but not significant in bivariate analysis.

Response: You have asked an interesting question. In our study, we assessed 36 variables using our data collection tools. Unfortunately, some variables were not found. For example, we assessed ‘’TB- comorbidity’’ in our investigation, but we did not find any TB-comorbidity case. In this sentence we want to indicate the number of variables assessed during data collection. We apologize for the expression '36 variables tested for bivariate analysis'. The number of variables tested were those listed in table1, table 2 and table 3. we hope that the edited section of our manuscript will clarifies this expression better. 

26. The statement: “Moreover, the likely hood of developing second line virologic failure among patients with Viral load >1000 copes/ml when switched to second line ART medication were 3.5 times (AOR=3.56, 95% CI: 1.5 - 8) more likely as compared to those patients who had Viral load result <1000 copes/ml”. Correction should be made in abstract, results and discussion section.It should be re-written as “…those with VL>1000 copies/ml had 3.5 times more to develop VF as compared to those with VL<150 copies /ml…” and ““…those with VL>150-999 copies/ml had 5 times more to develop VF as compared to those with VL<150 copies /ml…”

Response: We agree with your assessment and have incorporated this suggestion throughout our revised manuscript.

27. Discussion; Citation of reference should be at the end of a sentence, not in the middle of a sentence.

Response: Thank you for providing these insights. We have made a correction.

28. Acronym ‘…meds…’ do you mean medications. Correct it as required.

Response: Yes, it was to mean medications. We corrected it. 

29. Revise a statement “Double-edged sword of disclosure on ART adherence…”

You mentioned the advantage of disclosure on ART adherence, what was the disadvantage of disclosure on ART adherence.

Response: great look you did, thank you for this. We have elaborated the disadvantage of disclosure on ART adherence throughout our discussion in our revised manuscript.

30. Reference; It was referenced man

Attachment Submitted filename: Rebutal ltter.docx

# Peer review: Decision Letter 1

**DOI:** 10.1371/journal.pone.0289450.r003

**Authors:** Yoon-Seok Chung

20 Feb 2024

Title - Determinants of virological failure among HIV clients on second-line antiretroviral treatment at Felege-hiwot and University of Gondar comprehensive specialized hospitals in the Amhara region, Northwest Ethiopia: case-control study.

PONE-D-23-20506R1

Dear Dr. Getie,

We’re pleased to inform you that your manuscript has been judged scientifically suitable for publication and will be formally accepted for publication once it meets all outstanding technical requirements.

Within one week, you’ll receive an e-mail detailing the required amendments. When these have been addressed, you’ll receive a formal acceptance letter and your manuscript will be scheduled for publication.

An invoice for payment will follow shortly after the formal acceptance. To ensure an efficient process, please log into Editorial Manager at http://www.editorialmanager.com/pone/, click the 'Update My Information' link at the top of the page, and double check that your user information is up-to-date. If you have any billing related questions, please contact our Author Billing department directly at authorbilling@plos.org.

If your institution or institutions have a press office, please notify them about your upcoming paper to help maximize its impact. If they’ll be preparing press materials, please inform our press team as soon as possible -- no later than 48 hours after receiving the formal acceptance. Your manuscript will remain under strict press embargo until 2 pm Eastern Time on the date of publication. For more information, please contact onepress@plos.org.

Kind regards,

Yoon-Seok Chung

Academic Editor

PLOS ONE

Additional Editor Comments (optional):

Reviewers' comments:

Reviewer's Responses to Questions

**Comments to the Author**

1. If the authors have adequately addressed your comments raised in a previous round of review and you feel that this manuscript is now acceptable for publication, you may indicate that here to bypass the “Comments to the Author” section, enter your conflict of interest statement in the “Confidential to Editor” section, and submit your "Accept" recommendation.

Reviewer #1: All comments have been addressed

Reviewer #2: All comments have been addressed

**********

2. Is the manuscript technically sound, and do the data support the conclusions?

The manuscript must describe a technically sound piece of scientific research with data that supports the conclusions. Experiments must have been conducted rigorously, with appropriate controls, replication, and sample sizes. The conclusions must be drawn appropriately based on the data presented. 

Reviewer #1: Yes

Reviewer #2: Yes

**********

3. Has the statistical analysis been performed appropriately and rigorously? 

Reviewer #1: Yes

Reviewer #2: Yes

**********

4. Have the authors made all data underlying the findings in their manuscript fully available?

The PLOS Data policy requires authors to make all data underlying the findings described in their manuscript fully available without restriction, with rare exception (please refer to the Data Availability Statement in the manuscript PDF file). The data should be provided as part of the manuscript or its supporting information, or deposited to a public repository. For example, in addition to summary statistics, the data points behind means, medians and variance measures should be available. If there are restrictions on publicly sharing data—e.g. participant privacy or use of data from a third party—those must be specified.

Reviewer #1: Yes

Reviewer #2: Yes

**********

5. Is the manuscript presented in an intelligible fashion and written in standard English?

PLOS ONE does not copyedit accepted manuscripts, so the language in submitted articles must be clear, correct, and unambiguous. Any typographical or grammatical errors should be corrected at revision, so please note any specific errors here.

Reviewer #1: Yes

Reviewer #2: Yes

**********

6. Review Comments to the Author

Please use the space provided to explain your answers to the questions above. You may also include additional comments for the author, including concerns about dual publication, research ethics, or publication ethics. (Please upload your review as an attachment if it exceeds 20,000 characters)

Reviewer #1: All my comments and concerns are adrressed in this version. I checked and revier this version, the authors accept and correct . In this version no addtional comments and concern.

Reviewer #2: Corrections

. Language polishing is required.

Line-92: ...HIV ADIS, is it ...HIV AIDS..?

Line 182: crosschecked .. cross checked...?

Line 280: ..likely hood ... likelihood...

**********

7. PLOS authors have the option to publish the peer review history of their article (what does this mean?). If published, this will include your full peer review and any attached files.

If you choose “no”, your identity will remain anonymous but your review may still be made public.

**Do you want your identity to be public for this peer review?** For information about this choice, including consent withdrawal, please see our Privacy Policy.

Reviewer #1: **Yes: **Esubalew Tesfahun

Reviewer #2: **Yes: **Abilo Tadesse

**********

# Editor report: Acceptance letter

**DOI:** 10.1371/journal.pone.0289450.r004

**Authors:** Yoon-Seok Chung

28 Jun 2024

PONE-D-23-20506R1 

PLOS ONE

Dear Dr. Getie, 

I'm pleased to inform you that your manuscript has been deemed suitable for publication in PLOS ONE. Congratulations! Your manuscript is now being handed over to our production team.

At this stage, our production department will prepare your paper for publication. This includes ensuring the following:

* All references, tables, and figures are properly cited

* All relevant supporting information is included in the manuscript submission,

* There are no issues that prevent the paper from being properly typeset

If revisions are needed, the production department will contact you directly to resolve them. If no revisions are needed, you will receive an email when the publication date has been set. At this time, we do not offer pre-publication proofs to authors during production of the accepted work. Please keep in mind that we are working through a large volume of accepted articles, so please give us a few weeks to review your paper and let you know the next and final steps. 

Lastly, if your institution or institutions have a press office, please let them know about your upcoming paper now to help maximize its impact. If they'll be preparing press materials, please inform our press team within the next 48 hours. Your manuscript will remain under strict press embargo until 2 pm Eastern Time on the date of publication. For more information, please contact onepress@plos.org.

If we can help with anything else, please email us at customercare@plos.org.

Thank you for submitting your work to PLOS ONE and supporting open access. 

Kind regards, 

PLOS ONE Editorial Office Staff

on behalf of

Dr. Yoon-Seok Chung 

Academic Editor

PLOS ONE
